# Subjective–Device-Estimated Sleep Discrepancies in Late-Life Depression: Agreement and Associated Factors

**DOI:** 10.31083/AP50350

**Published:** 2026-06-30

**Authors:** Qianlong Wang, Yuandong Gong, Jiacui Ji, Yan Zhang, Meixu Lu, Meiyu Song, Xia Zhang, Yuping Zhao, Qi Miao

**Affiliations:** ^1^School of Mental Health, Jining Medical University, 272075 Jining, Shandong, China; ^2^Shandong Mental Health Center, Shandong University, Shandong Provincial Key Medical and Health Discipline of Gerontology, 250014 Jinan, Shandong, China

**Keywords:** aged, major depressive disorder, monitoring, sleep duration, self report

## Abstract

**Background:**

This study examined the agreement between subjective sleep assessments and device-estimated sleep parameters in patients with late-life depression, and explored potential factors associated with the discrepancies between the two measures.

**Methods:**

This cross-sectional study enrolled 94 patients with late-life depression. Device-estimated sleep parameters were obtained via portable sleep respiratory monitoring. Participants also reported the previous night’s total sleep time (TST), sleeponset latency (SOL), and sleep efficiency (SE) using a self-designed form. Agreement between subjective and device-estimated measures was evaluated with Bland–Altman analysis. Multivariate regression was conducted to identify factors associated with subjective–device-estimated sleep discrepancies.

**Results:**

Compared with device-estimated measures, subjective reports differed significantly across all three sleep parameters (Wilcoxon signed-rank test: TST, *Z* = −6.94, *p* < 0.001; SOL, *Z *= −2.98, *p *= 0.003; SE, *Z *= −5.02, *p* < 0.001). These results indicate a pronounced subjective–device-estimated sleep discrepancy in late-life depression, with subjective underestimation of TST and SE and overestimation of SOL. Greater depressive symptom severity was associated with greater subjective underestimation of TST (*p* = 0.037), whereas later age at first onset was associated with greater subjective underestimation of SE (*p* = 0.013). The association between age at first onset and subjective overestimation of SOL observed in the main analysis (*p* = 0.047) was no longer significant after additional inclusion of sex in the model (*p* = 0.054).

**Conclusions:**

Patients with late-life depression exhibited subjective–device-estimated discrepancies in sleep parameters. Heavier depressive symptom burden was linked to greater underestimation of total sleep time, while later age at first onset was more closely related to underestimation of sleep efficiency. A later age at first onset showed a trend toward an association with overestimation of sleep onset latency, but this finding still requires further validation. In clinical practice, combining device-estimated sleep monitoring with subjective sleep assessment should be considered in order to obtain a more comprehensive understanding of patients’ sleep status.

## Main Points

1. Patients with late-life depressive disorder exhibited subjective–device-estimated sleep discrepancies.

2. Agreement between self-reported and device-estimated sleep was poor for total sleep time (TST), sleep onset latency (SOL), and sleep efficiency (SE).

3. Greater depressive symptom severity (17-item Hamilton Depression Rating Scale, HAMD-17) was associated with a larger underestimation of sleep duration, suggesting that a higher symptom burden may exacerbate subjective–device-estimated sleep discrepancy.

4. A later age at first onset was associated with greater underestimation of SE and greater overestimation of SOL in the main analysis, although the latter association was not retained after additional adjustment for sex.

## 1. Introduction

Sleep is a fundamental physiological process essential for maintaining human health, and untreated sleep disturbances are associated with a range of adverse health outcomes [1]. Approximately 50% of older adults report difficulties in initiating or maintaining sleep [2]. The relationship between depression and sleep disturbance is likely bidirectional. About 60% of individuals with depression experience sleep problems [3]. Depressive disorder is a risk factor for the onset of insomnia, and insomnia increases the subsequent risk of developing depression by more than fourfold [4]. Previous research has also indicated that sleeping fewer than 7 hours per night is associated with unfavorable health consequences, including weight gain and obesity, diabetes, hypertension, heart disease and stroke, depression, and increased mortality risk [5]. Late-life depression refers to major depressive disorder that occurs in adults aged 60 years or older [6]. It is associated with substantial disease burden, disability, functional decline, elevated risks of suicide and mortality, and patients frequently report sleep disturbances [7]. Sleep quality is an important component of quality-of-life assessment in late-life depression and plays a critical role in maintaining cognitive function and reducing the risk of dementia in older adults [8]. Therefore, accurate assessment of sleep quality is of significant clinical importance for understanding and improving sleep problems in patients with late-life depression.

Sleep quality can be assessed using a variety of approaches, including subjective measures [9] and objective measures [10]. However, in the context of illness, older adults’ subjective sleep reports may be biased due to age-related memory decline [11]; depression-related negative cognitive bias may also affect their recall and appraisal of sleep duration and quality [12]. Therefore, relying solely on patients’ self-reports to evaluate sleep quality may be inaccurate in clinical practice.

In recent years, increasing attention has been paid to the agreement between subjective sleep perceptions and objectively measured sleep outcomes. When an individual’s perceived sleep substantially diverges from results obtained through objective assessment, this phenomenon is often termed “sleep state misperception” [13] in the insomnia literature; however, in this study we use the term descriptively to denote subjective–device-estimated discrepancies, rather than as an International Classification of Sleep Disorders (ICSD)-based diagnostic classification. Among older adults with comorbid mild cognitive impairment and subthreshold depression, approximately 61% have been reported to exhibit discrepancies between subjective and objective sleep measures [11]. Subjective–objective sleep discrepancy is also commonly observed in patients with major depressive disorder [14]. Studies have shown that, in individuals with depressive disorders, subjective sleep reports often differed substantially from objectively measured sleep parameters [15], suggesting that patients’ self-evaluations of sleep may be unreliable. Such subjective–objective discrepancies are also frequent in the general older population. In a community-based study of older adults [9], comparisons between subjective sleep indices derived from the Pittsburgh Sleep Quality Index (PSQI) and the Consensus Sleep Diary (CSD) and objective indices obtained via actigraphy indicated marked differences between perceived sleep quality and objectively assessed sleep status. Another study reported that, in questionnaire-based assessments, older adults rated their sleep efficiency (SE) as poorer than that obtained through objective evaluation [16]. In studies of patients with insomnia, many individuals demonstrated misperceptions of their sleep; most tended to underestimate total sleep time (TST) and overestimate sleep onset latency (SOL) [14,17,18,19].

Previous studies have documented subjective–objective differences in sleep indices among adults with depression, individuals with insomnia, and older adults without psychiatric disorders. Moreover, studies across different populations have examined potential factors associated with such discrepancies. For example, subjective sleep estimates in adults with depression have been linked to objective sleep parameters, depression severity, age, and personality traits [20]. Among adults with bipolar disorder, depressive symptoms have been associated with greater misperception of TST [21]. In addition, research has suggested that greater sleep fragmentation and affective dysregulation in patients with insomnia may contribute to discrepancies between subjective and objective sleep measures [16]. In the general population, anxiety-related traits such as repetitive negative thinking and poor stress-coping capacity may contribute to underestimation of sleep duration [22]. Among older adults with mild cognitive decline and subthreshold depression, slowed memory processing has been reported as a factor associated with subjective–objective sleep discrepancies [11].

However, most previous studies have focused on adults with depression, patients with insomnia, or older adults without major psychiatric disorders; evidence specific to late-life depression remains limited. Cognitive impairment is more common in patients with late-life depression [23], with particularly poorer performance in processing speed and working memory [24]. Other studies have shown that cognitive deficits in late-life depression are closely related to vascular dysfunction and overall vascular risk burden. Specifically, the Framingham vascular risk score has been associated with impairments in processing speed, executive function, and episodic memory [25]. In addition, executive or memory impairment, together with a higher cerebrovascular-disease burden, may increase the likelihood that an individual’s sleep recall and time estimation deviate from their actual sleep status.

Multiple cross-sectional studies have shown that sleep quality is closely associated with a range of metabolic and endocrine indicators. Sleep duration has been linked to an increased risk of dyslipidemia, including hypercholesterolemia [26], hyperlipidemia [27], and elevated low-density lipoprotein cholesterol (LDL-C) and reduced high-density lipoprotein cholesterol (HDL-C) levels [28,29]. One study reported a significant negative correlation between free triiodothyronine (T3) levels and sleep duration among individuals sleeping ≤7 hours [30]. In patients with thyroid abnormalities, individuals with subclinical hypothyroidism had higher PSQI scores than did those with normal thyroid function [31]. In a study examining the association between sleep duration and the risk of hyperhomocysteinemia, the relationship exhibited a U-shaped pattern, with both insufficient and excessive sleep associated with a greater risk of hyperhomocysteinemia [32]. Another study found that short sleep duration may be associated with higher serum homocysteine levels [33]. In a study on the associations of uric acid with sleep quality and sleep duration, poor sleep quality was associated with lower uric acid levels, whereas short sleep duration was associated with higher uric acid levels [34]; additionally, lower serum uric acid levels have been linked to chronic insomnia and its severity [35]. Therefore, metabolic and vascular risk–related markers such as dyslipidemia and elevated homocysteine may increase bias in individuals’ subjective estimations of total sleep time, nocturnal awakenings, and sleeponset latency. Additionally, fluctuations in thyroid function and a stress-axis–related hyperarousal state may amplify nocturnal somatic vigilance and the sense of wakefulness [36], leading to worse subjective sleep experiences. Based on this rationale, the present study included laboratory indices such as triglycerides (TG), total cholesterol (CHOL), homocysteine (HCY), uric acid (UA), and thyroid function as potential correlates in exploratory analyses, in order to provide clues to the biological mechanisms underlying subjective–device-estimated sleep discrepancies in patients with late-life depressive disorder.

This study focused on three distinct sleep dimensions—TST, SE, and SOL. Among these, TST is a core indicator of sleep quality, directly reflecting sleep adequacy and serving as an important predictor of both physical and mental health [37]. SE reflects the ease of falling asleep and returning to sleep after nocturnal awakenings [38], and lower SE has been associated with increased risks of mortality [39], cardiovascular disease [40], and diabetes [41]. Prolonged SOL has been associated with increased risks of dementia in older adults [42], higher mortality risk [42], cardiovascular disease [43] and metabolic syndrome [44]. Shorter SOL has been linked to a reduced risk of stroke [43]. Based on these considerations, we compared the agreement between subjective and device-estimated sleep measures across TST, SE, and SOL, and further explored potential factors associated with subjective–device-estimated discrepancies, with the aim of informing the clinical evaluation of sleep problems in patients with late-life depression.

## 2. Materials and Methods

### 2.1 Participants

Eligible participants were newly admitted inpatients with late-life depression at the Shandong Mental Health Center between January 2024 and September 2025, who were screened according to predefined inclusion and exclusion criteria. The inclusion criteria were as follows: (1) meeting the diagnostic criteria for depressive disorder according to the International Classification of Diseases, 10th Revision (ICD-10); (2) age ≥60 years, with at least one depressive episode occurring at or after the age of 60 years; (3) Han Chinese ethnicity, with no restrictions on sex; (4) no systematic antidepressant treatment within 2 weeks prior to enrollment (“no systematic antidepressant treatment” means that participants had not continuously used prescribed antidepressant medications before enrollment, and stable maintenance antidepressant therapy was not permitted) and no modified electroconvulsive therapy (MECT) within 3 months prior to enrollment; and (5) ability to cooperate with the required assessments and laboratory examinations.

To minimize potential confounding from other psychiatric or medical conditions, the exclusion criteria were: (1) depression secondary to organic pathology or other psychiatric disorders; (2) severe organic brain disease or other serious physical illnesses; (3) severe violent or suicidal behaviors that precluded completion of assessments or examinations; and (4) comorbid intellectual disability, dementia, epilepsy, or terminal illness.

A total of 94 patients with late-life depression were ultimately included. This study was approved by the Ethics Committee of Shandong Mental Health Center, and written informed consent was obtained from all participants prior to enrollment.

### 2.2 Instruments and Measures

Sociodemographic and clinical characteristics were collected using a self-designed questionnaire, including sex, age, years of education, and age at first onset.

#### 2.2.1 Portable Sleep Respiratory Monitoring (Device-Estimated Sleep Parameters)

Device-estimated sleep parameters were obtained using the PSM100A portable sleep respiration monitor (Sealand Technology, Chengdu, China). The device integrates nasal airflow, pulse oximetry (SpO_2_), single-lead electrocardiogram (ECG), thoracic bio-impedance, snoring, body position, and body movement, and generates an automated analysis report based on multimodal signal fusion and cardiopulmonary coupling (CPC) -related analysis. CPC-based approaches can be computed from reduced signals such as a single-lead ECG or photoplethysmography (PPG) by extracting heart rate variability and ECG/PPG-derived respiration, enabling portable applications [45]. Data were analyzed using the manufacturer’s software (PSM, v. 1.1, Sealand Technology). The PSM100A has previously been applied in clinical samples that included older adults [46]. Monitoring was performed as whole-night recording, and the automatically generated report was reviewed/verified by a physician for quality control. SOL was defined as the interval from recording start to device-detected sleep onset. TST was the total duration scored as sleep by the device during the recording. SE was calculated as TST / recording duration (test time) × 100%. In this study, “objective” referred to portable-monitor–derived (device-estimated) sleep parameters rather than polysomnography (PSG)-based electroencephalography (EEG) sleep staging.

#### 2.2.2 Subjective Sleep Assessment

Subjective sleep parameters for the night of portable monitoring were obtained the next morning using a brief self-designed sleep report form. This form was adopted to maintain feasibility in the inpatient setting and had not undergone formal piloting or validation. Participants were asked to report their best estimates according to clock time and their own sleep experience. We chose this brief form to obtain same-night subjective estimates that were closely aligned, in timing and content, with the device-estimated TST, SOL, and time in bed. For newly admitted older inpatients with depression, this approach was also more manageable than longer questionnaires intended to assess habitual sleep over longer recall periods.

The form included items assessing: (1) bedtime and get-up time (to derive time in bed), (2) SOL (in min), defined as the estimated time from attempting to fall asleep to sleep onset, and (3) TST (in min), defined as the estimated total amount of sleep during the night. Subjective SE was calculated as TST/time in bed × 100%, where time in bed was defined as the interval between bedtime and get-up time.

#### 2.2.3 Depressive and Anxiety Symptoms

These symptoms and their severity were assessed using the 17-item Hamilton Depression Rating Scale (HAMD-17) and the Hamilton Anxiety Rating Scale (HAMA), with higher scores indicating more severe symptoms. Subjective cognitive function was evaluated using the Perceived Deficits Questionnaire-Depression (PDQ-D).

#### 2.2.4 Laboratory Measures

After an overnight fast, peripheral venous blood samples were collected between 06:30 and 07:00 the following day and immediately sent to the Department of Clinical Laboratory at the Shandong Mental Health Center for analysis. TG, CHOL, HDL-C, LDL-C, small dense low-density lipoprotein cholesterol (sd LDL-C), lipoprotein (Lp), HCY, and UA were measured using an AU5800 automated biochemical analyzer. Thyroid-stimulating hormone (TSH), T3, thyroxine (T4), free thyroxine (FT4), and free triiodothyronine (FT3) were determined using a Mindray CL6000i chemiluminescence immunoassay analyzer (Mindray Bio-Medical Electronics Co., Ltd., Shenzhen, China). All assays were performed by experienced laboratory personnel in strict accordance with the manufacturers’ instructions and standard operating procedures.

### 2.3 Statistical Analysis

All statistical analyses were performed using SPSS (v. 27.0, IBM Corp., Armonk, NY, USA). Systematic differences between the two measurement methods were evaluated using the Wilcoxon signed-rank test. We performed normality testing using the Shapiro–Wilk test. For Bland–Altman analyses, parametric limits of agreement (mean difference ± 1.96 SD) were reported when the subjective–device-estimated differences were approximately normally distributed, whereas a non-parametric (percentile-based) Bland–Altman approach (median difference with the 2.5th and 97.5th percentile limits) was used when normality was violated. Bland–Altman agreement analyses and plots were generated using MedCalc version 23.3.4 (MedCalc Software Ltd, Ostend, Belgium). Bland–Altman plots provide a graphical assessment of agreement between two methods; agreement was considered poor when the 95% limits of agreement (LoA) exceeded the predefined acceptable range. In addition, agreement was evaluated by determining whether the maximum difference within the limits of agreement was clinically acceptable. As in previous work [47], we predefined clinically acceptable ranges for the differences between subjective and device-estimated measures. Specifically, the following *a priori* interpretive criteria were applied to the mean bias and LoA: ±30 min for TST; ±15 min for SOL; and ±10% for SE. Proportional bias was assessed by linear regression of the differences (subjective − device-estimated) on the mean of the two methods (difference-on-mean regression). A statistically significant slope indicated that discrepancies varied with the magnitude of the measurement. This analysis was performed in MedCalc version 23.3.4 (MedCalc Software Ltd). In addition, the proportion of (subjective − device-estimated) differences falling within the predefined clinically acceptable range was calculated as a quantitative index of agreement.

For univariable analyses, the Shapiro–Wilk test was used to assess the normality of continuous variables. Pearson correlation was applied to normally distributed continuous data, whereas Spearman correlation was used for continuous variables with non-normal distributions. Categorical variables were analyzed using nonparametric methods, and between-group comparisons were conducted with the Mann–Whitney U test. All tests were two-sided, and *p *< 0.05 was considered statistically significant. Univariable analyses were exploratory; thus, nominal (unadjusted) *p* values were reported and should be interpreted cautiously given multiple comparisons.

To identify potential factors associated with subjective–device-estimated sleep discrepancies, multivariable linear regression analyses were performed. Given the exploratory aim and limited sample size, we used univariable screening (*p* < 0.05) to select predictors for parsimonious multivariable models. However, this approach may potentially miss variables that remain important in multivariable models [48].

To complement *p*-values, standardized effect sizes were reported: effect size r (|Z|/ $\sqrt{N}$ ) for Wilcoxon signed-rank tests, Pearson’s r / Spearman’s r_s_ for correlations, and standardized β coefficients for multivariable linear regression.

To address potential correlation between HAMD-17 and HAMA, multicollinearity was assessed using tolerance/variance inflation factors (VIF) and collinearity diagnostics. Linear-regression assumptions were evaluated using residual diagnostics (histogram and normal P–P plot for normality; residuals-versus-fitted plots for linearity and homoscedasticity). Influence statistics (e.g., Cook’s distance) were examined; when an influential observation was identified, sensitivity analyses were performed by refitting the model after excluding the most influential case. Given that missing data were confined to a small number of laboratory variables and occurred infrequently, multiple imputation was not undertaken. Available-case data were used for descriptive and univariable analyses, while multivariable regression models were estimated from complete cases for the variables entered into each model. To evaluate the possibility of bias related to missingness, participants with and without missing laboratory data were compared in terms of age and sex using the Mann–Whitney U test and Fisher’s exact test, respectively. Given the female predominance in the sample, additional sensitivity analyses were performed by forcing sex into each multivariable regression model, irrespective of its univariable association with the outcome, to assess the robustness of the main findings to sex adjustment.

## 3. Results

### 3.1 Participant Characteristics

Table 1 presents the demographic and clinical characteristics, biological markers, and subjective and device-estimated sleep parameters of the study sample. A total of 94 patients with late-life depression were included, with a mean age of 70.55 ± 5.95 years, and 74.47% were female.

**Table 1.** **General characteristics of participants, biological markers, and overall subjective and device-estimated sleep parameters**.

Variables	*n*	Mean (SD) or percentage
Sex		
Male	24	25.53%
Female	70	74.47%
Age (years)	94	70.55 ± 5.95
Years of education	94	7.11 ± 4.80
Age at first onset	94	62.59 ± 11.17
HAMA score	94	22.81 ± 7.94
HAMD-17 score	94	20.10 ± 7.02
PDQ-D score	94	38.63 ± 15.68
TG (mmol/L)	94	1.26 ± 0.58
CHOL (mmol/L)	94	4.76 ± 2.42
HDL-C (mmol/L)	94	1.34 ± 0.28
LDL-C (mmol/L)	94	2.55 ± 0.92
sd LDL-C (mmol/L)	94	0.90 ± 0.31
Lp (a) (mg/L)	94	228.29 ± 237.78
HCY (μmol/L)	93	12.00 ± 3.49
UA (μmol/L)	94	273.34 ± 88.30
T3 (ng/mL)	92	0.80 ± 0.14
T4 (μg/dL)	92	8.10 ± 1.67
FT3 (pg/mL)	92	2.53 ± 0.40
FT4 (ng/dL)	92	1.39 ± 0.29
TSH (μIU/mL)	92	2.29 ± 3.51
Subjective TST (min)	94	320.74 ± 129.32
Device-estimated TST (min)	94	452.84 ± 83.97
Subjective SOL (min)	94	70.9 ± 55.01
Device-estimated SOL (min)	94	50.73 ± 39.95
Subjective SE (%)	94	62.73 ± 21.83
Device-estimated SE (%)	94	77.69 ± 9.51

Note. Data are presented as mean (SD). Sex is presented as n (%). Missing data were limited to a few laboratory measures because blood samples were not collected in a small number of participants; therefore, sample sizes vary slightly across these variables (HCY, n = 93; T3, T4, FT3, FT4, and TSH, n = 92 each). Analyses were performed using available-case data. “Device-estimated” sleep parameters were derived from portable sleep respiratory monitoring. TST, total sleep time; SOL, sleep onset latency; SE, sleep efficiency; HAMD-17, 17-item Hamilton Depression Rating Scale; HAMA, Hamilton Anxiety Rating Scale; PDQ-D, Perceived Deficits Questionnaire-Depression; TG, Triglycerides; CHOL, total cholesterol; HDL-C, high-density lipoprotein cholesterol; LDL-C, low-density lipoprotein cholesterol; sd LDL-C, small dense low-density lipoprotein cholesterol; Lp, lipoprotein; HCY, homocysteine; UA, uric acid; TSH, thyroid-stimulating hormone; T3, triiodothyronine; T4, thyroxine; FT3, free triiodothyronine; FT4, free thyroxine.

Missing data were limited to a few laboratory measures and were attributable to missed blood collection in a small number of participants. The main sleep variables were complete for all 94 participants. Participants with and without missing laboratory data did not differ significantly in age (Mann–Whitney U = 74.5, Z = –1.336, *p* = 0.181) or sex (Fisher’s exact test, two-sided *p* = 1.000).

### 3.2 Differences Between Subjective and Device-Estimated Sleep Parameters

Table 2 presents the subjective and device-estimated measures of TST, SOL, and SE. Compared with device-estimated measures, subjective reports differed significantly across all three sleep parameters: patients underestimated TST and SE, but overestimated SOL (Wilcoxon signed-rank test: TST, Z = −6.94, *p* < 0.001, r = 0.72; SOL, Z = −2.98, *p* = 0.003, r = 0.31; SE, Z = −5.02, *p* < 0.001, r = 0.52). The mean biases (subjective minus device-estimated) were −132.10 ± 144.38 min for TST, 20.17 ± 67.22 min for SOL, and −14.97 ± 24.70% for SE.

**Table 2.** **Subjective and portable sleep respiratory monitoring measurements of TST, SOL, and SE (*n* = 94)**.

Variable	Self-reported sleep measures recorded using a self-designed form		Portable sleep respiratory monitoring		Bias	*Z*	*p*
	Mean (SD)	±95% CI	Mean (SD)	±95% CI			
TST (min)	320.74 ± 129.32	294.26~347.23	452.84 ± 83.97	435.64~470.04	–132.10 ± 144.38	–6.939	<0.001
SOL (min)	70.90 ± 55.01	59.64~82.17	50.73 ± 39.95	42.55~58.92	20.17 ± 67.22	–2.979	0.003
SE (%)	62.73 ± 21.83	58.256~67.20	77.69 ± 9.51	75.74~79.64	–14.97 ± 24.70	–5.023	<0.001

Notes. Mean bias = subjective − device-estimated. Effect size: r = |Z| / $\sqrt{N}$ (Z, standardized Wilcoxon statistic; *n*, number of paired observations).

### 3.3 Agreement Between Subjective and Device-Estimated Measures

Normality checks of the Bland–Altman differences showed that the TST difference (subjective–device-estimated) was approximately normally distributed (W = 0.981, *p* = 0.184), whereas the SOL difference (W = 0.909, *p *< 0.001) and the SE difference (W = 0.964, *p* = 0.010) deviated from normality.

#### 3.3.1 Agreement for TST (Bland–Altman Analysis)

**Fig. 1.**
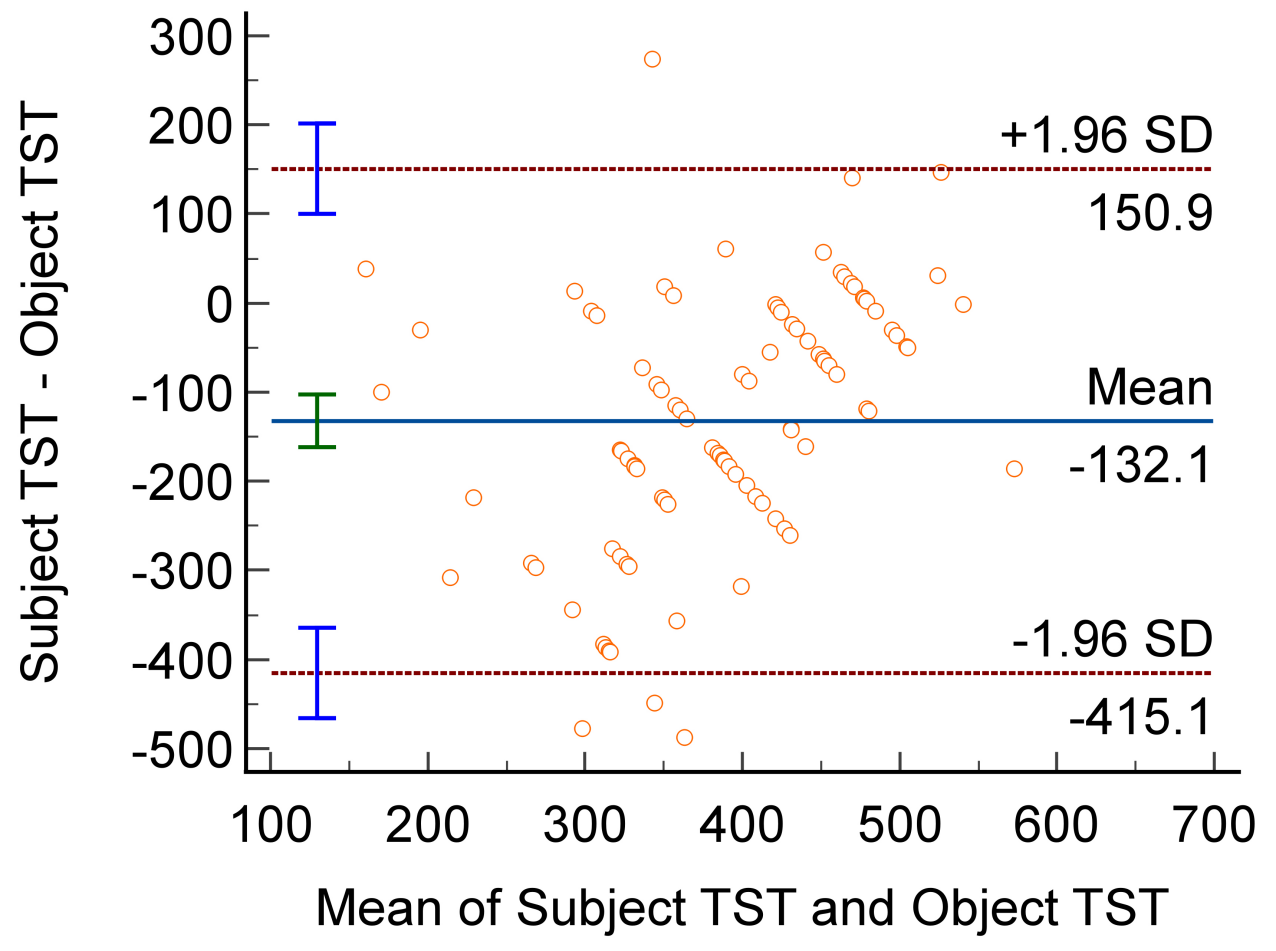
**Bland–Altman plot comparing subjective TST recorded using the self-designed form with TST measured by the portable sleep respiratory monitoring device. **Bias: −132.1 min (95% CI: −161.7 to −102.5 min). 95% limits of agreement (LoA): −415.1 to 150.9 min (Lower LoA 95% CI: −465.8 to −364.4 min; Upper LoA 95% CI: 100.2 to 201.6 min).

Differences were calculated as subjective TST minus device-estimated TST and plotted against the mean of the two methods. The mean bias was −132.1 min, indicating that patients tended to underestimate TST by approximately 2.2 h. The 95% LoA (−415.1 to 150.9 min) clearly exceeded the predefined clinically acceptable range (±30 min), suggesting poor agreement between subjective and device-estimated TST (Fig. 1).

The TST discrepancy (subjective − device-estimated) ranged from −487 to 274 min, indicating substantial inter-individual variability. Outliers were evaluated using the **interquartile range (**IQR) rule (Q1 = −218.8 min; Q3 = −13.4 min; IQR = 205.4 min; fences: −526.8 to 294.7 min), and no outliers were identified (0 cases), suggesting that the observed heterogeneity was not driven by extreme values.

#### 3.3.2 Agreement for SOL (Bland–Altman Analysis)

**Fig. 2.**
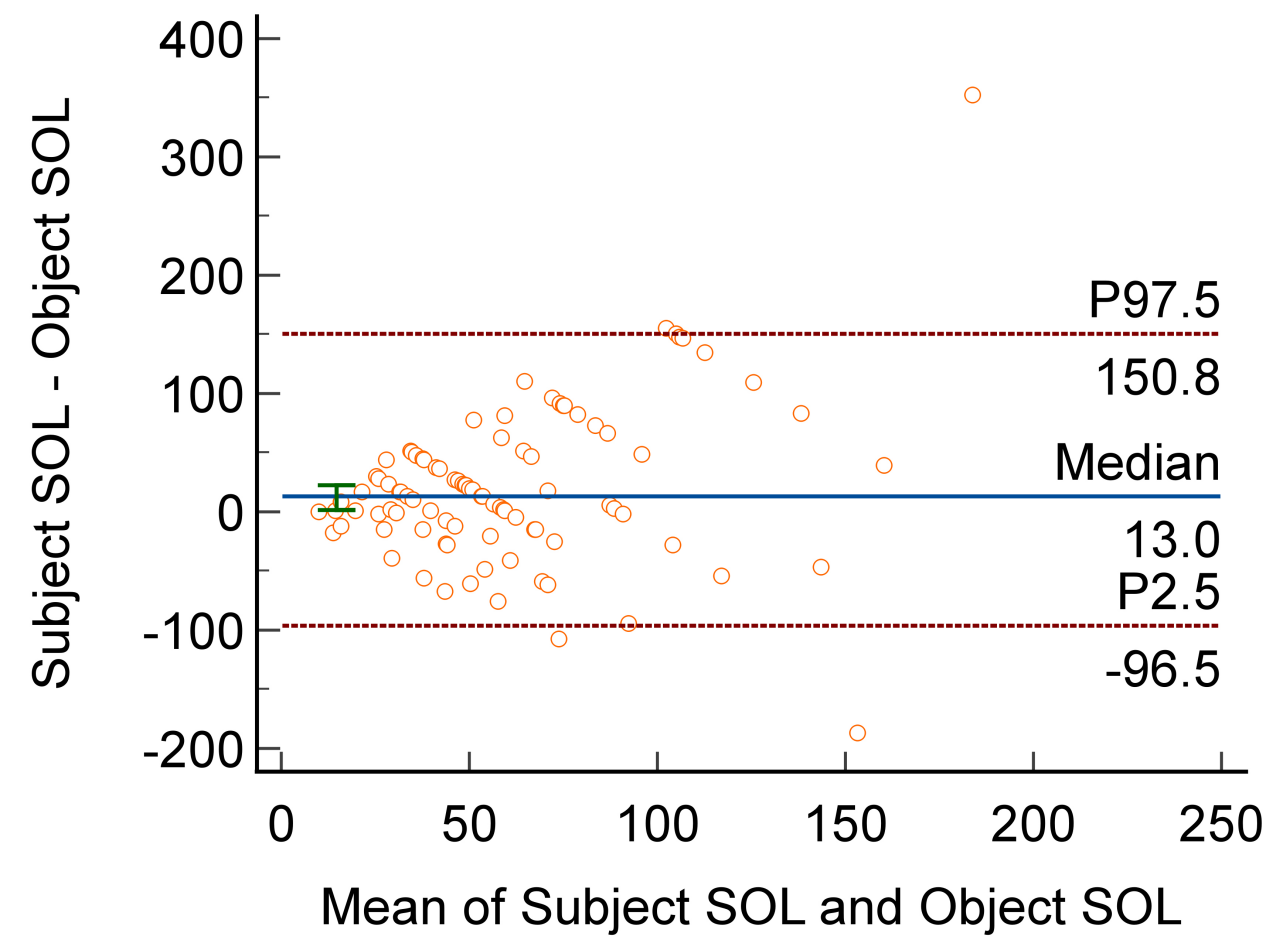
**Non-parametric Bland–Altman plot of differences (subjective SOL − device-estimated SOL) versus the mean of the two methods**. Median difference (bias): 13.0 min (95% CI: 1.5 to 22.5 min). Percentile limits of agreement (P2.5–P97.5): −96.5 min to 150.8 min.

Using the non-parametric (percentile-based) Bland–Altman method, the limits of agreement for subjective versus device-estimated SOL (−96.5 to 150.8 min) clearly exceeded the predefined clinically acceptable range (±15 min), indicating poor agreement between self-reported SOL and SOL derived from the portable sleep respiratory monitoring device (Fig. 2).

#### 3.3.3 Agreement for SE (Bland–Altman Analysis)

**Fig. 3.**
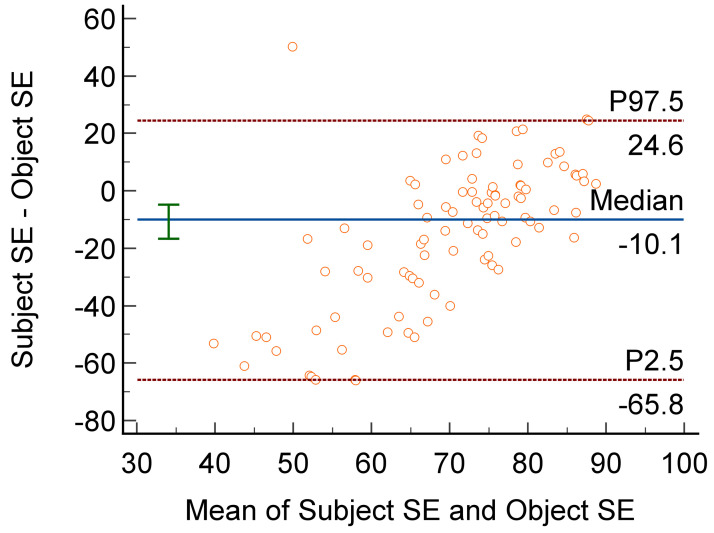
**Non-parametric Bland–Altman plot of differences (subjective SE − device-estimated SE) versus the mean of the two methods**. Median difference (bias): −10.1% (95% CI: −16.7% to −4.8%). Percentile limits of agreement (P2.5–P97.5): −65.8% to 24.6%.

Using the non-parametric (percentile-based) Bland–Altman method, the limits of agreement for subjective versus device-estimated SE (−65.8% to 24.6%) clearly exceeded the predefined clinically acceptable range (±10%), indicating poor agreement between self-reported SE and SE derived from the portable sleep respiratory monitoring device (Fig. 3).

In addition to reporting the bias and limits of agreement, we further calculated quantitative agreement metrics. Under the predefined clinically acceptable thresholds (±30 min for TST, ±15 min for SOL, and ±10% for SE), only 20.2% (TST), 30.9% (SOL), and 37.2% (SE) of participants had differences within the acceptable range, indicating poor clinical agreement. To assess proportional bias, we performed linear regression of the differences on the mean of the two methods. Proportional bias was observed for TST (slope = 0.7244, *p* < 0.0001), SOL (slope = 0.6052, *p* = 0.0024), and SE (slope = 1.4744, *p* < 0.0001), suggesting that discrepancies varied systematically with the magnitude of the measurements (Figs. 1,2,3).

### 3.4 Univariable Analyses of Factors Associated With Sleep Discrepancies

#### 3.4.1 Factors Associated With TST Discrepancy (Subjective − Device-Estimated)

These findings indicated that the subjective–device-estimated TST discrepancy was negatively associated with the severity of anxiety and depressive symptoms as well as with subjective cognitive function, and positively associated with HCY levels (Table 3).

**Table 3.** **Univariable analyses of associations between sociodemographic and clinical characteristics and the sleep time discrepancy (subjective − device-estimated)**.

Variables	*n*	Test	*p*
Sex		*Z *= –1.474	0.140
Male	24		
Female	70		
Age (years)	94	r = 0.086	0.411
Years of education	94	r_s_ = 0.138	0.184
Age at first onset	94	r_s_ = –0.194	0.061
HAMA score	94	r = –0.387	<0.001
HAMD-17 score	94	r = –0.388	<0.001
PDQ-D score	94	r = –0.269	0.009
TG (mmol/L)	94	r_s_ = –0.092	0.378
CHOL (mmol/L)	94	r_s_ = –0.097	0.355
HDL-C (mmol/L)	94	r_s_ = –0.083	0.429
LDL-C (mmol/L)	94	r_s_ = –0.129	0.215
sd LDL-C (mmol/L)	94	r = –0.084	0.420
Lp (a) (mg/L)	94	r_s_ = 0.092	0.377
HCY (μmol/L)	93	r_s_ = 0.232	0.025
UA (μmol/L)	94	r_s_ = 0.159	0.126
T3 (ng/mL)	92	r = –0.024	0.817
T4 (μg/dL)	92	r_s_ = –0.060	0.568
FT3 (pg/mL)	92	r_s_ = 0.062	0.558
FT4 (ng/dL)	92	r_s_ = –0.011	0.920
TSH (μIU/mL)	92	r_s_ = –0.051	0.628

Notes. For categorical variables, the Mann–Whitney U test was used, and the standardized statistic is reported as *Z*.

The discrepancy in total sleep time (subjective − device-estimated) was significantly correlated with the HAMA score (r = –0.387, *p *< 0.001), HAMD-17 score (r = –0.388, *p *< 0.001), HCY (r_s_ = 0.232, *p *= 0.025), and PDQ-D score (r = –0.269, *p *= 0.009).

#### 3.4.2 Factors Associated With SOL Discrepancy (Subjective − Device-Estimated)

The discrepancy in sleep onset latency (subjective − device-estimated) was significantly correlated with the HAMA score (r_s_ =0.341, *p* < 0.001), HAMD-17 score (r_s_ = 0.255, *p *= 0.013), age at first onset (r_s_ = 0.255, *p *= 0.013), and PDQ-D score (r_s_ = 0.26, *p *= 0.011). These results indicated that the subjective–device-estimated SOL discrepancy was positively associated with the severity of anxiety and depressive symptoms, subjective cognitive impairment, and age at first onset (Table 4).

**Table 4.** **Univariable analyses of associations between sociodemographic and clinical characteristics and the sleep onset latency discrepancy (subjective − device-estimated)**.

Variables	*n*	Test	*p*
Sex		*Z *= –0.507	0.612
Male	24		
Female	70		
Age (years)	94	r_s_ = –0.031	0.767
Years of education	94	r_s_ = –0.077	0.459
Age at first onset	94	r_s_ = 0.255	0.013
HAMA score	94	r_s_ = 0.341	<0.001
HAMD-17 score	94	r_s_ = 0.255	0.013
PDQ-D score	94	r_s_ = 0.260	0.011
TG (mmol/L)	94	r_s_ = –0.012	0.907
CHOL (mmol/L)	94	r_s_ = –0.003	0.979
HDL-C (mmol/L)	94	r_s_ = 0.022	0.834
LDL-C (mmol/L)	94	r_s_ = 0.017	0.873
sd LDL-C (mmol/L)	94	r_s_ = –0.001	0.992
Lp (a) (mg/L)	94	r_s_ = –0.052	0.618
HCY (μmol/L)	93	r_s_ = –0.104	0.322
UA (μmol/L)	94	r_s_ = –0.043	0.684
T3 (ng/mL)	92	r_s_ = –0.017	0.871
T4 (μg/dL)	92	r_s_ = 0.076	0.471
FT3 (pg/mL)	92	r_s_ = –0.111	0.294
FT4 (ng/dL)	92	r_s_ = 0.023	0.830
TSH (μIU/mL)	92	r_s_ = 0.131	0.215

#### 3.4.3 Factors Associated With SE Discrepancy (Subjective − Device-Estimated)

The discrepancy in sleep efficiency (subjective − device-estimated) was significantly correlated with the HAMA score (r_s_ = –0.314, *p *= 0.002), HAMD-17 score (r_s_ = –0.244, *p* = 0.018), age at first onset (r_s_ = –0.267, *p *= 0.009), and thyroid stimulating hormone TSH(r_s_ = –0.22, *p *= 0.035). These findings indicate that the subjective–device-estimated SE discrepancy was negatively associated with the severity of anxiety and depressive symptoms, age at first onset, and TSH levels (Table 5).

**Table 5.** **Univariable analyses of associations between sociodemographic and clinical characteristics and the sleep efficiency discrepancy (subjective − device-estimated)**.

Variables	*n*	Test	*p*
Sex		*Z *= –1.565	0.118
Male	24		
Female	70		
Age (years)	94	r_s_ = –0.062	0.556
Years of education	94	r_s_ = 0.180	0.082
Age at first onset	94	r_s_ = –0.267	0.009
HAMA score	94	r_s_ = –0.314	0.002
HAMD-17 score	94	r_s_ = –0.244	0.018
PDQ-D score	94	r_s_ = –0.075	0.471
TG (mmol/L)	94	r_s_ = –0.076	0.467
CHOL (mmol/L)	94	r_s_ = –0.113	0.278
HDL-C (mmol/L)	94	r_s_ = –0.029	0.781
LDL-C (mmol/L)	94	r_s_ = –0.165	0.113
sd LDL-C (mmol/L)	94	r_s_ = –0.142	0.172
Lp (a)(mg/L)	94	r_s_ = 0.188	0.070
HCY (μmol/L)	93	r_s_ = 0.090	0.390
UA (μmol/L)	94	r_s_ = 0.083	0.427
T3 (ng/mL)	92	r_s_ = –0.130	0.216
T4 (μg/dL)	92	r_s_ = –0.137	0.194
FT3 (pg/mL)	92	r_s_ = –0.085	0.419
FT4 (ng/dL)	92	r_s_ = 0.006	0.954
TSH (μIU/mL)	92	r_s_ = –0.220	0.035

### 3.5 Multivariate Regression Analyses

Using the TST discrepancy (subjective − device-estimated) as the dependent variable, the HAMA total score, HAMD-17 total score, HCY, and PDQ-D score were entered as independent variables in a multivariate linear regression model. The model was statistically significant (adjusted R^2^ = 0.187, F = 6.303, *p* < 0.001). The HAMD-17 total score remained associated with the TST discrepancy (B = −5.10, SE = 2.41, standardized β = −0.246, *p *= 0.037). Each 1-point increase in HAMD-17 was associated with an additional 5.1-min underestimation of TST (subjective − device-estimated).

Using the SOL discrepancy (subjective − device-estimated) as the dependent variable, the HAMA total score, HAMD-17 total score, age at first onset, and PDQ-D score were entered as independent variables in a multivariate linear regression model. The model was statistically significant (adjusted R^2^ = 0.071, F = 2.770, *p *= 0.032). Age at first onset remained associated with the SOL discrepancy (B = 1.225, SE = 0.608, standardized β = 0.204, *p *= 0.047), indicating an approximately 1.2-min increase in SOL overestimation per 1-year increase in age at first onset.

Using the SE discrepancy (subjective − device-estimated) as the dependent variable, the HAMA total score, HAMD-17 total score, age at first onset, and TSH were entered as independent variables in a multivariate linear regression model. The model was statistically significant (adjusted R^2^ = 0.140, F = 4.719, *p *= 0.002). Age at first onset remained associated with the SE discrepancy (B = −0.556, SE = 0.219, standardized β = −0.249, *p *= 0.013), indicating an additional 0.56-percentage-point underestimation of SE per 1-year increase in age at first onset (Table 6).

**Table 6.** **Multivariate linear regression analyses of subjective–device-estimated discrepancies (subjective − device-estimated) in TST, SOL, and SE**.

Independent variable	Unstandardized coefficients		Standardized coefficients	*t*	*p*	VIF
	B	SE	β			
TST discrepancy						
HAMD-17 total score	–5.100	2.407	–0.246	–2.119	0.037	1.532
HAMA total score	–3.645	2.556	–0.199	–1.426	0.157	2.215
PDQ-D	–0.783	1.085	–0.085	–0.722	0.472	1.573
HCY	6.813	3.932	0.164	1.733	0.087	1.014
SOL discrepancy						
age at first onset	1.225	0.608	0.204	2.014	0.047	1.024
HAMD-17 total score	0.341	1.190	0.036	0.286	0.775	1.546
HAMA total score	1.938	1.257	0.229	1.541	0.127	2.209
PDQ-D	0.017	0.528	0.004	0.032	0.975	1.576
SE discrepancy						
age at first onset	–0.556	0.219	–0.249	–2.537	0.013	1.018
HAMD-17 total score	–0.530	0.429	–0.151	–1.238	0.219	1.582
HAMA total score	–0.662	0.378	–0.213	–1.752	0.083	1.566
TSH	–0.699	0.714	–0.099	–0.980	0.33	1.085

**Notes**. Discrepancies were defined as subjective − device-estimated. B, unstandardized coefficient; SE, standard error; β, standardized coefficient; VIF, variance inflation factor. Model fit statistics: TST model, adjusted R^2^ = 0.187, F = 6.303, *p* < 0.001; SOL model, adjusted R^2^ = 0.071, F = 2.770, *p* = 0.032; SE model, adjusted R^2^ = 0.140, F = 4.719, *p* = 0.002.

Among the candidate variables entered, only the HAMD-17 total score was significant in the final adjusted model for the TST discrepancy (standardized β ≈ −0.25), suggesting a more stable relative contribution. For the SOL and SE discrepancy models, only age at first onset was retained in the final models (standardized β ≈ 0.20 and −0.25, respectively).

For the TST discrepancy model, multicollinearity was not problematic (tolerance 0.452–0.986; VIF 1.014–2.215; maximum condition index 13.509), and residual diagnostics did not indicate major violations (Durbin–Watson = 1.606; standardized residuals min −2.928, max 2.974; maximum Cook’s distance = 0.275). For the SOL discrepancy model, multicollinearity was not problematic (tolerance 0.453–0.977; VIF 1.024–2.209; maximum condition index 18.567), and residual diagnostics did not indicate major violations (Durbin–Watson = 1.385; standardized residuals min −3.524, max 4.696; maximum Cook’s distance = 0.253). For the SE discrepancy model, one highly influential observation was identified in the full sample (maximum Cook’s distance = 2.515); therefore, a sensitivity analysis excluding the most influential case (caseid = 89; *n* = 91) was performed. In the sensitivity model, multicollinearity remained acceptable (tolerance 0.621–0.975; VIF 1.026–1.611; maximum condition index 18.224) and residual diagnostics did not suggest major violations (Durbin–Watson = 1.862; standardized residuals min −2.094, max 3.134; maximum Cook’s distance = 0.066). The sensitivity model remained significant (F = 5.225, *p* < 0.001; adjusted R^2^ = 0.158), and age at first onset remained significant (B = −0.598, *p* = 0.008).

As women made up most of the sample, we further repeated the multivariable analyses after adding sex to each model. Sex was not significantly related to TST discrepancy (*p* = 0.386), SOL discrepancy (*p* = 0.884), or SE discrepancy (*p* = 0.159). After adjustment for sex, the overall pattern of results remained broadly similar to that of the primary analyses: the association between HAMD-17 total score and TST discrepancy remained significant (*p* = 0.035), and age at first onset remained associated with SE discrepancy (*p* = 0.008), whereas the finding for SOL discrepancy was no longer statistically significant once sex was included in the model (*p* = 0.054).

## 4. Discussion

Our findings indicated systematic biases between subjective and device-estimated sleep measures in patients with late-life depression. Specifically, patients tended to overestimate sleep onset latency and underestimate total sleep time and sleep efficiency, suggesting that they perceived their sleep quality as poorer than that indicated by device-estimated monitoring. These results indicate marked subjective–device-estimated sleep discrepancies in late-life depression. Multivariable regression analyses indicated that greater depressive symptom severity was associated with greater underestimation of total sleep time. Age at first onset was associated with underestimation of sleep efficiency and with overestimation of sleep onset latency in the primary model (although the latter association was no longer statistically significant after additional adjustment for sex).

### 4.1 Key Characteristics of Subjective–Device-Estimated Sleep Discrepancies in Late-Life Depression

Our results indicated that patients with late-life depression exhibited discrepancies between subjective and device-estimated sleep assessments, characterized by subjective overestimation of sleep-onset latency and underestimation of TST and SE. Similar discrepancies are common among patients with insomnia. Previous studies have shown that individuals with insomnia often overestimate sleeponset latency and underestimate TST and SE, whereas polysomnography or actigraphy indicated that their sleep quality is not as poor as they perceived [49]. Our findings were consistent with that pattern. Previous research suggested that subjective–objective sleep discrepancies in insomnia primarily arise from the interaction between sleep instability (e.g., increased light sleep and a higher density of cortical microarousals) and impaired emotion regulation. This interaction may amplify patients’ subjective perception of sleep fragmentation, thereby exacerbating the gap between subjective and objective sleep assessments [16]. Other studies have shown that patients with primary insomnia tend to overestimate sleeponset latency, and this sleep misperception is associated with elevated β-band EEG activity during sleep initiation [50]. One study reported subjective–objective sleep discrepancies among older adults with mild cognitive impairment and subthreshold depression, and these discrepancies were associated with memory impairment [11]. Previous studies have shown that cognitive arousal is associated with subjective–objective discrepancies in total sleep time, and an experimental increase in cognitive arousal can further enlarge this discrepancy [51]. That phenomenon may be related to arousal-induced distortion of time perception: when the brain processes more information per unit time, the same objective duration may be experienced as longer [51]. In addition, some evidence has suggested that elevated cognitive arousal during sleep onset induces heightened sensory and memory processing, blurring the boundary between sleep and wakefulness, and thereby exacerbating discrepancies between subjective and objective assessments [52]. Some studies have further proposed that subjective–objective sleep discrepancies are associated with central nervous system hyperarousal [53].

Taken together, our findings suggested that patients with late-life depression tended to overestimate sleeponset latency and underestimate total sleep time and sleep efficiency, indicating marked discrepancies between subjective sleep perception and device-estimated monitoring results. Based on previous evidence, we speculate that these discrepancies may be related to mechanisms such as sleep fragmentation, increased cortical microarousals, and heightened cognitive arousal; however, further basic and mechanistic studies are needed to explore and validate these hypotheses. Clinically, our results also suggested that relying solely on subjective reports may lead to misjudgment of the severity of sleep problems in patients with late-life depression, and that it may be useful to integrate device-estimated sleep monitoring with subjective assessments for a more comprehensive evaluation. It should be emphasized that the multivariable models explained a relatively small proportion of the outcome variance (particularly for SOL); therefore, the correlates identified in this study should be regarded as exploratory signals and require replication in independent samples. The modest adjusted R^2^ values likely reflect the multifactorial nature of subjective–device-estimated sleep discrepancies in late-life depression and the fact that several potentially relevant factors, such as objective cognitive performance, vascular or neuroimaging burden, medication-related variables, and night-to-night variability, were not included in this study.

### 4.2 Association Between Depression Severity and Underestimation of Subjective Total Sleep Time

In this study, the total HAMD-17 score was associated with underestimation of sleep duration in patients with late-life depression, suggesting that greater depressive symptom severity was linked to a larger subjective underestimation of total sleep time. However, evidence on the association between depression severity and underestimation of sleep duration remains limited, and findings across studies have been inconsistent. Many potential factors that may have influenced this association have not yet been fully investigated. One study reported that, among adults with depression, subjective and objective sleep duration were correlated and that the magnitude of their discrepancy was associated with depression severity [20]. By further stratifying participants into underestimation and overestimation groups, those authors found that the extent of the subjective–objective difference in total sleep time was significantly related to depressive severity, and that patients in the TST-underestimation group had more severe depressive symptoms. Those findings suggested that depressive symptoms are linked to sleep misperception and that patients with depression tended to estimate their sleep inaccurately, with the degree of inaccuracy influenced by depression severity — consistent with our results. However, most related studies were conducted in adult samples with depression, whereas the present study focused on patients with late-life depression. Given age-specific physiological and cognitive changes in later life, the manifestation of subjective–objective sleep discrepancies and their potential correlates may differ in this population. By further examining the association between depressive symptom severity and subjective–objective sleep discrepancies, our findings suggested that late-life factors may play an important role in the development of biased sleep perception. In a community-based study of older adults [54], depressive symptom levels were associated with reports of poorer subjective sleep quality, whereas no association was found between depressive symptom levels and total sleep time. Those findings suggested that depressive symptoms may have primarily influenced individuals’ subjective sleep experience rather than objective sleep duration. Results of study of patients with insomnia showed that, among those with normal objective total sleep time, anxiety-related rumination and insufficient stress-coping resources may have mediated the underestimation of total sleep time [22]. Studies have also shown that, in patients with late-life depression, greater depression severity was an important contributor to cognitive impairment and was associated with slower information processing speed and with memory decline [55]. We hypothesized that when cognitive and memory abilities are impaired, patients are more likely to emphasize their subjective experience of nocturnal wakefulness or difficulty sleeping when recalling the entire night. This tendency may make individuals with more severe depressive symptoms more prone to subjectively underestimating total sleep time; however, this hypothesis warrants further investigation and validation.

### 4.3 Association Between Age at First Onset and Misestimation of Sleep Onset Latency and Sleep Efficiency

In the present study, among patients with late-life depression, a later age at first onset was associated with greater underestimation of SE and greater overestimation of SOL in the primary model (the association with SOL was no longer statistically significant after additional adjustment for sex).

These findings suggested that, in late-life depression, a later age at first onset may be linked to more pronounced subjective–device-estimated sleep discrepancies; however, this association should be interpreted as correlational rather than causal. Age at first onset may be regarded as a coarse clinical marker of heterogeneity in late-life depression.

The vascular depression hypothesis posits that cerebrovascular pathology may increase vulnerability to, precipitate, or perpetuate late-life depressive syndromes and is associated with related neurobiological changes [56]. Neuroimaging studies have shown that, compared with early-onset depression, late-onset late-life depression more commonly exhibits subcortical or deep white-matter hyperintensities and related structural alterations, suggesting a greater vascular burden and more pronounced abnormalities in fronto–subcortical networks [57].

Mechanistically, such neurovascular and circuit-level changes may be linked to cognitive vulnerabilities commonly observed in late-life depression (e.g., executive dysfunction, attentional deficits/slowed information-processing speed, and memory impairment). These cognitive processes may influence sleep recall and time estimation, making patients more likely to perceive their sleep experience as “worse than objective indicators”, thereby manifesting as subjective overestimation of SOL and subjective underestimation of SE [58]. In addition, subjective–device-estimated sleep discrepancies are generally considered multifactorial and may involve combined effects of arousal level and information-processing biases, rather than being attributable to a single mechanism [19].

Because the present study did not include neuroimaging markers of vascular burden, systematic quantification of vascular risk factors, detailed illness-course variables, or objective cognitive assessments, the above interpretations require further validation.

To date, few studies in late-life depression have examined whether age at first onset is related to subjective–device-estimated discrepancies in SOL and SE. Our findings more consistently supported a relationship with SE discrepancy, whereas the finding for SOL discrepancy was less robust after sex adjustment. These results still add to the understanding of subjective–device-estimated sleep incongruence in late-life depression.

### 4.4 Limitations

The present study focused on patients with late-life depression, systematically characterizing the agreement between subjective and device-estimated sleep parameters and further examining the associations between clinical factors (e.g., depression severity and age at first onset) and subjective–device-estimated sleep discrepancies. Several limitations should be noted: (1) this study was cross-sectional; therefore, causal inferences cannot be drawn; (2) subjective sleep was assessed using a self-designed, non-validated instrument and was based on a single-night report, which may have introduced measurement bias related to recall, item interpretation, and limited reliability, thereby affecting the precision of subjective TST, SOL, and SE estimates. In addition, the device-estimated sleep parameters were device-estimated from portable monitoring rather than derived from PSG/EEG-based sleep staging. (3) Predictors were selected for entry into the multivariable models based on univariable significance screening, which may have omitted potentially relevant variables and reduced model stability, and no adjustment for multiple comparisons was applied; thus, the regression findings should be regarded as exploratory. Moreover, model explanatory power was limited, as indicated by modest adjusted R^2^ values. Although sex-adjusted analyses produced findings broadly similar to those of the primary models, the association between age at first onset and SOL discrepancy was attenuated after sex adjustment, indicating that this finding may be less robust. Some residual confounding from other demographic variables may still remain. Moreover, the higher proportion of women in the sample means that the results may better reflect female patients with late-life depression and should be generalized to male patients with caution. (4) Sleep-active medications used around the monitoring night (type/dose/timing) were not systematically quantified; therefore, residual confounding due to medication effects cannot be fully excluded. Although recent antidepressant exposure before enrollment was controlled for, prior medication history and other treatment-related factors may still have contributed to residual confounding. Cognitive function was assessed using a self-report measure (PDQ-D) rather than objective testing, and thus shared method bias may be present. (5) Moreover, because this was a relatively small, single-center study, the sample may not fully represent the broader population of patients with late-life depression, and the findings may not fully extend to other clinical settings.

Future studies should use sleep diaries and conduct consecutive multi-night monitoring assessments, ideally complemented by polysomnography, to improve the comparability and accuracy of device-estimated sleep evaluation and to reduce first-night effects. Future work should apply multiple-comparison correction and replicate the findings in larger samples, where possible. In addition, sleep-active medications and antidepressant regimens during the monitoring period (type, dose, and timing) should be quantified more systematically and, when sample size permits, included as covariates in the models. Objective cognitive testing should also be incorporated to mitigate the impact of shared self-report bias on the results.

## 5. Conclusions

In this study of hospitalized patients with late-life depression, we systematically evaluated the agreement between subjective and device-estimated sleep parameters (TST, SOL, and SE) and explored potential factors associated with subjective–device-estimated discrepancies. Our findings indicated that patients with late-life depression tend to overestimate sleep onset latency and underestimate total sleep time and sleep efficiency. Moreover, depressive symptom severity and age at first onset appeared to be associated with these discrepancies, with a less robust finding for SOL after sex adjustment. Overall, this study highlighted pronounced subjective–device-estimated sleep incongruence in late-life depression and provided preliminary evidence linking depression severity and age at first onset to subjective–device-estimated sleep discrepancy.

## Data Availability

The data supporting the conclusions of this study are available from the corresponding author upon reasonable request.
